# Sanguinarine–Chelerythrine from *Coptis chinensis* Offers Analgesic and Anti-Inflammatory Effects Without Gastrotoxicity

**DOI:** 10.3390/pharmaceutics17030323

**Published:** 2025-03-02

**Authors:** Maciej Danielewski, Sylwia Zielińska, Anna Merwid-Ląd, Marta Szandruk-Bender, Wojciech Słupski, Maciej Włodarczyk, Tomasz Sozański, Piotr Ziółkowski, Adam Szeląg, Beata Nowak

**Affiliations:** 1 Department of Pharmacology, Wroclaw Medical University, ul. J. Mikulicza-Radeckiego 2, 50-345 Wroclaw, Poland; maciej.danielewski@umw.edu.pl (M.D.); anna.merwid-lad@umw.edu.pl (A.M.-L.); marta.szandruk-bender@umw.edu.pl (M.S.-B.); wojciech.slupski@umw.edu.pl (W.S.); adam.szelag@umw.edu.pl (A.S.); 2 Department of Pharmaceutical Biotechnology, Wroclaw Medical University, ul. Borowska 211, 50-556 Wroclaw, Poland; sylwia.zielinska@umw.edu.pl; 3 Department of Pharmacognosy and Herbal Medicines, Wroclaw Medical University, ul. Borowska 211A, 50-556 Wroclaw, Poland; maciej.wlodarczyk@umw.edu.pl; 4 Department of Preclinical Sciences, Pharmacology and Medical Diagnostics, Faculty of Medicine, Wroclaw University of Science and Technology, Wybrzeze Wyspianskiego 27, 50-370 Wroclaw, Poland; tomasz.sozanski@pwr.edu.pl; 5 Department of Pathology, Wroclaw Medical University, ul. K. Marcinkowskiego 1, 50-368 Wroclaw, Poland; piotr.ziolkowski@umw.edu.pl

**Keywords:** sanguinarine, chelerythrine, *Coptis chinensis*, analgesic activity

## Abstract

**Background**: Pain is a major clinical and socioeconomic problem worldwide. The available therapies are not always effective and are often associated with the multiple adverse effects that reduce their clinical application. Natural compounds are an important group of pharmaceuticals that may be used in pain management. We aimed to investigate the analgesic activity of the sanguinarine–chelerythrine from *Coptis chinensis*. **Methods**: The analgesic and anti-inflammatory activity of the sanguinarine–chelerythrine fraction of *C. chinensis* extract (SC 5 and 10 mg/kg), sanguinarine (SAN 1 and 2 mg/kg) and chelerythrine (CHEL 4 and 8 mg/kg) was assessed in tail flick and formalin tests. A microscopic and macroscopic examination of stomach mucosae was performed. TNFα and MMP-9 levels were measured with ELISA kits. **Results**: Morphine (MORF), CHEL and SC prolongated the tail withdrawal latency, with comparable analgesic activity between MORF and CHEL 8 mg/kg. MORF, CHEL 8 mg/kg, and SAN 2 mg/kg ameliorated the pain reaction in the neurogenic phase of the formalin test. In the inflammatory phase of the formalin test, all tested substances exerted analgesic activity. SAN, CHEL and SC additionally reduced TNFα and MMP-9 secretion. **Conclusions**: Our results confirmed analgesic effects of CHEL and SC with CHEL analgesic activity comparable to MORF. All investigated substances exerted significant anti-inflammatory activity without concomitant gastrotoxicity.

## 1. Introduction

Pain is a major clinical and socioeconomic problem worldwide [1,2,3]. The International Association for the Study of Pain (IASP) defines pain as “an unpleasant sensory and emotional experience associated with, or resembling that associated with, actual or potential tissue damage” [4]. Pain significantly impacts individuals’ quality of life and increases costs to the health system. Additionally, chronic pain belongs to the leading causes of disability and disease burden globally [5]. It is important to bear in mind that untreated or ineffectively treated pain is associated with an increased prevalence of comorbidities. Chronic persistent pain has been reported to be, among others, a contributing factor to the reduction in cognitive function, insomnia occurrence, sexual dysfunction, and various depressive and anxiety disorders [6]. However, existing analgesic therapies are not always effective and are often accompanied with the various adverse effects that reduce their clinal utility. Nonsteroidal anti-inflammatory drugs (NSAIDs) are very efficient in the treatment of inflammatory pain, but gastrointestinal side effects often restrict their administration. Opioid drugs are another effective analgesic option; however, although they do not increase the risk of gastric bleeding, their long-term administration is associated with a significant risk of drug abuse. Therefore, it is extremely important to search for new safer therapeutic options, and natural compounds are an important group of pharmaceuticals that may be used in pain management.

Rhizomes of *Coptis chinensis* are known in Chinese pharmacopoeia as *Coptidis Rhizoma* (CR) or Huang Lian [7]. CR belongs to one of the most important components of many traditional Chinese formulations that have been used in the treatment of diarrhea, other gastrointestinal disorders, diabetes, toothache, inflammatory disorders, fever, and skin diseases [8]. Shengyang Sanhuo Decoction containing CR is prescribed in traditional Chinese medicine, among others, in the treatment of neuropathic pain [9], and its effectiveness is attributed to the inhibition of TNFα, IL-6, and CRP production. Various contemporary studies confirmed the anti-inflammatory [10,11,12] and analgesic [7] activity of *C. chinensis*. However, most studies have focus on berberine as the most abundant isoquinoline alkaloid present in CR [13,14]. CR contains over 100 various chemical constituents, including, among others, berberine, sanguinarine, and chelerythrine, which are the main bioactive ingredients [8]. It has been suggested that the analgesic activity of CR may be attributed to the inhibition of TRPV1 (transient receptor potential cation channel subfamily V member 1) and NF-κB (nuclear factor kappa-light-chain-enhancer of activated B cells) overexpression and the activation of the JNK/p38 MAPK (c-Jun N-terminal kinase/p38 mitogen-activated protein kinase) pathways [9]. However, further studies investigating the activity of individual constituents are needed.

**Figure 1:**
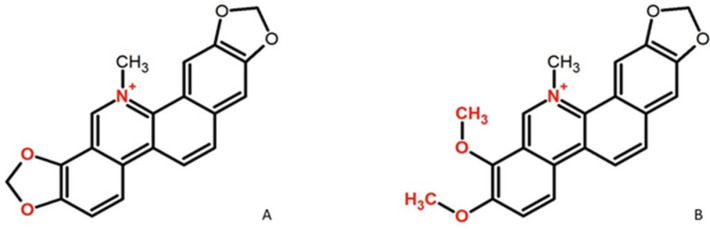
Chemical structure of investigated alkaloids. (**A**) Sanguinarine, (**B**) chelerythrine.

Alkaloids are nitrogen-containing chemical compounds that are found in plants, and several investigations have demonstrated their various biological activity. Alkaloids found in CR can be divided into several subgroups according to their structures. Berberine, the most abundant alkaloid, is a protoberberine derivative, whereas sanguinarine and chelerythrine are benzophenanthridine (BZD) quaternary amine alkaloids (Figure 1).

Plants producing BZDs have played an important role in traditional folk medicine for centuries. Both sanguinarine and chelerythrine have demonstrated anti-inflammatory, anticancer, and antiviral activity in various studies [15,16]. It has also been reported that sanguinarine interferes with nerve impulse transmission due to its inhibitory effects on choline acetyltransferase activity and its hindering of nicotinic, muscarinic, and serotonin receptors activity [15]. Some authors reported also that sanguinarine may ameliorate neuropathic pain [17,18,19]. Although the chemical structure of sanguinarine and chelerythrine is similar, it was reported that they exert different effects on glycine transporters [20], which are promising targets for analgesic therapy. Therefore, it seems to be of great relevance to compare the analgesic activity of both alkaloids.

In our previous study, we demonstrated the anti-inflammatory activity of the sanguinarine–chelerythrine fraction of *C. chinensis* extract in a carrageenan paw edema test [21]. The current work is a continuation of our previous study, and therefore, the doses investigated in the reported study were based on our previous results [21]. In the reported study, we aimed to investigate the analgesic activity of the sanguinarine–chelerythrine fraction of *C. chinensis* extract and broaden the knowledge on the activity of both alkaloids by comparison of the analgesic and anti-inflammatory activity of isolated constituents and their mixture. To our knowledge, this is the first experiment to compare the analgesic and anti-inflammatory activity of *C. chinensis* extract and the two alkaloid components administered separately. We aimed to answer the question of whether the natural mixture of alkaloids from the extract has a stronger or weaker effect than the corresponding doses of substances administered separately and to assess the dose–effect dependency. Another novelty of the study lies in the fact that apart from the assessment of anti-inflammatory activity, analgesic effect was also investigated.

## 2. Materials and Methods

### 2.1. Chemicals and Materials

We used in the experiment the following chemicals and drugs: sanguinarine chloride (purity (HPLC) ≥ 90%) (Extrasynthese, Genay, France) (SAN); chelerythrine chloride (purity (HPLC) ≥ 95%) (Extrasynthese, Genay, France) (CHEL); morphine sulphate 10 mg/mL (Morphini Sulfas WZF ^®^, Zaklady Farmaceutyczne Polpharma, Starogard Gdanski, Poland); indomethacin (purity (HPLC) ≥ 98%) (Sigma-Aldrich, Steinheim, Germany); 0.9% saline solution (Zaklady Farmaceutyczne Polpharma, Starogard Gdanski, Poland); xylazine 20 mg/mL (Sedazin^®^, Biowet, Pulawy, Poland); ketamine 100 mg/mL (Biowet, Pulawy, Poland); and formalin 37% sol. (Chempur, Piekary Slaskie, Poland). Other used chemicals were included in the commercially available kits.

### 2.2. Plant Compound Preparation

The fraction of *C. chinensis* extract containing sanguinarine and chelerythrine (SC) was administered to rats in tail flick and formalin tests. The investigated fraction was isolated as a mixture (0.2:1 *w*/*w*) from *C. chinensis* rhizoma (19 g/100 g yield) as described previously [22]. The UHPLC proof of composition and purity of fraction CS was performed using the Thermo Scientific UltiMate 3000 system (Thermo Scientific, Waltham, MA, USA), coupled with a 4. detector controlled by DataAnalysis software version 4.2 (Bruker Daltonics, Germany). The analyses were performed in positive mode in the range 120–1400 *m*/*z*.

The analytical column was Halo Phenyl-Hexyl 2.7 µm, 2.1 mm × 150 mm (AMT, Laurens, SC, USA). The solvents of LCMS purity (Merck, Germany) were 0.1% (*V*/*V*) formic acid solutions in water (A) and in MeCN (B). Each analysis was calibrated with sodium formate, run at 30 °C, and the solvent flow was 0.3 mL/min. The composition of the mobile phase was as follows (% B in A (min)): 2(0) → 2(1) → 100(31) → 100(35.5) → 2(37) → 2(40).

**Figure 2:**
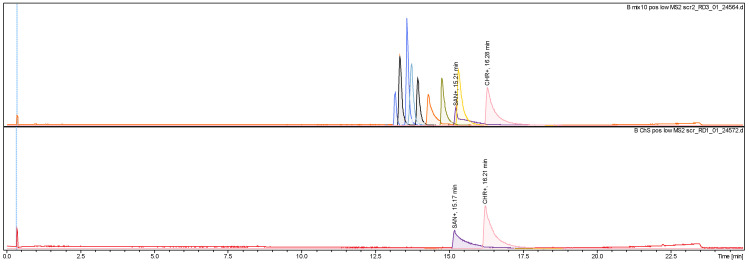
LCMS chromatogram of investigated alkaloid fraction (**below**) against a set of alkaloid standards at equivalent concentrations (**above**). Sanguinarine (SAN+; C_20_H_14_NO_4_^+^ 332.0925 *m*/*z*, err. −2.2 ppm; Rt 15.17 min) and chelerythrine (CHR+; C_21_H_18_NO_4_^+^ 348.124 *m*/*z*, err. −1.9 ppm; Rt 16.21 min).

**Figure 3:**
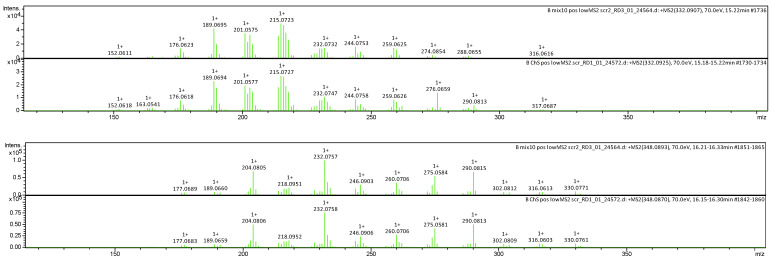
Sets of MS/MS comparisons of standard (**above**) vs. fraction constituent (**below**). Sanguinarine first, chelerythrine next.

Using a mixture of alkaloid standards, incl. sanguinarine and chelerythrine, the identity of the constituents and the composition of the investigated fraction was confirmed by a comparison between MS, MS/MS and Rt, as shown at Figure 2 and Figure 3. Using a set of dilutions of standards, it was estimated that sanguinarine and chelerythrine in this fraction were at a 1:1 ratio (m/m).

### 2.3. Animals

One hundred male Wistar rats (weighing 171.9 ± 22.5 g) purchased from the Animal Research Centre at Wroclaw Medical University (Wroclaw, Poland) were used in the study. The rats were housed in pairs in transparent polypropylene cages under standard conditions of temperature (21–23 °C), humidity (60–70%), and a light–dark cycle (12:12 h). The animals received a standard rodent diet (LSM, Agropol, Motycz, Poland). Access to food and water was ad libitum.

### 2.4. Drug Administration

**Figure 4:**
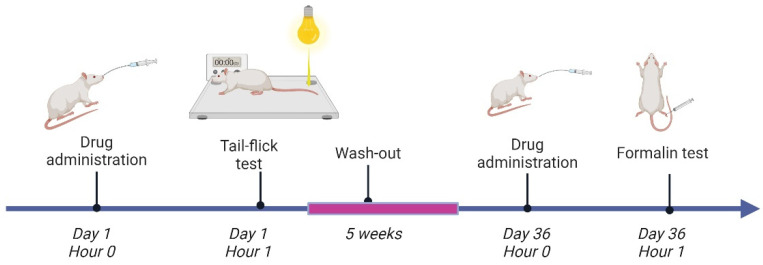
An outline of the experimental protocol. On Days 1 and 36, the animals receive the investigated substances 1 h prior to the tail flick test and the formalin test, respectively.

After acclimatization, the rats were randomly assigned to ten experimental groups, ten animals each: two control groups (a negative control group CON and a positive control group FOR) receiving 0.9% saline solution intragastrically (i.g.) (3 mL/kg), two groups receiving a fraction of *C. chinensis* containing sanguinarine and chelerythrine (5 and 10 mg/kg i.g. in saline solution 3 mL/kg) marked as SC5 and SC10, respectively, two groups receiving sanguinarine (1 and 2 mg/kg i.g. in saline solution 3 mL/kg) named SAN1 and SAN2, respectively, two groups receiving chelerythrine (4 and 8 mg/kg i.g. in saline solution 3 mL/kg) named CHEL4 and CHEL8, respectively, and two groups receiving reference drugs: indomethacin (IND) (10 mg/kg i.g. in saline solution 3 mL/kg) and morphine (MORF) (10 mg/kg i.g. in saline solution 3 mL/kg). The randomization process took into account the body weight to ensure the comparable mean body weight between the experimental groups. The doses of the investigated fraction of *C. chinensis* extract were established based on a previously reported study investigating the anti-inflammatory properties of the extract [21]. The administered doses of sanguinarine and chelerythrine were calculated as equivalents of their content in the corresponding doses of the investigated fraction of *C. chinensis* extract. The investigated substances were administered intragastrically one hour before the tail flick or formalin tests, as shown in Figure 4. There was a five-week-long wash-out period between both tests.

### 2.5. Tail Flick Test

We evaluated the antinociceptive effects of investigated substances with a tail flick apparatus (TF-01, Porfex, Bialystok, Poland). One hour after the administration of the appropriate experimental substance (saline solution in the negative control group, morphine in the group receiving a reference drug, and sanguinarine, chelerythrine or the investigated fraction of *C. chinensis* extract containing sanguinarine and chelerythrine in the experimental groups), the animal was briefly restrained with the hand, with its tail extended in a slot of variable width supplied with a groove that guarantees a precise placement and enables free movement. A heat stimulus of fixed intensity was applied to the cutaneous skin of the mid-tail (4.0–6.0 cm from the tip of the tail), and the time required for the animal to flick its tail from the stimulus was recorded. The tail flick latency was defined as the time (in seconds) for the rat to withdraw its tail from the radiant heat source. The cut-off time was 10 s to prevent tissue damage [23]. The result was blindly assessed by an experienced investigator.

The percentage of the analgesic activity was calculated with the following formula:

(1) $$\%\;analgesic\;activity=100\times \frac{tail\;withdrawal\;latency-basic\;latency}{cut\;off\;tail\;withdrawal\;latency-basic\;latency}$$

In the above formula, the basic latency was defined as the average tail withdrawal latency in the control group receiving saline solution.

### 2.6. Formalin Test

One hour after the administration of the appropriate experimental substance (saline solution in negative and positive control groups, morphine and indomethacin in the reference groups, and sanguinarine, chelerythrine or the investigated fraction of *C. chinensis* extract containing sanguinarine and chelerythrine in the experimental groups), 50 µL of 5% formalin (the positive control group, reference groups receiving FOR and IND, and all animals receiving investigated substances) or 50 µL of saline solution (the negative control group—CON) was injected subcutaneously into the dorsal surface of the right hind paw using a microsyringe with a 26-gauge needle. In total, 5% formalin solution was obtained by dilution of the commercially available 37% formalin solution with normal saline solution. The duration of licking, flinching, shaking, and biting (in seconds) of the injected paw during the neurogenic (early; 0–5 min) and inflammatory (late; 25–30 min) phase of the nociceptive reaction was measured as an expression of pain reaction. Two independent blinded observers measured the time of pain reaction was using a handheld stopwatch.

### 2.7. Isolation of Right Hind Paw

After euthanasia, the right hind paws were immediately cut off. The swollen tissue was isolated and homogenized and the obtained supernatant was frozen for a further enzyme-linked immunosorbent assay (ELISA). The concentration of tumor necrosis factor alpha (TNFα), and matrix metalloproteinase 9 (MMP-9) in the supernatant was assessed with commercial ELISA Kits (Nori Rat TNF alfa ELISA Kit, Genorise Scientific Inc., Glen Mills, PA, USA; and Nori Rat MMP-9 ELISA Kit, Genorise Scientific Inc., Glen Mills, PA, USA, respectively), according to the manufacturer’s instructions, with the BioTek Epoch ELISA system (Agilent Technologies, Santa Clara, CA, USA).

### 2.8. Macro- and Microscopic Examination of Gastric Mucosa

After euthanasia, the abdomen was opened and the stomach was excised. The removed stomach was rinsed with 5 mL of distilled water after being opened along greater curvature. In total, 0.1 M phosphate saline buffer (1:4 (*w/v*), pH 7.4) was used to preserve stomach tissues. Then, the stomach was fixed in 4% buffered formalin. Later, the stomach was embedded in paraffin and cut into 4 µm thick slices. The obtained stomach specimens were stained by the routine hematoxylin–eosin (H&E) method on the glass slides.

The macro- and microscopic examination allowed us to assess the damage of gastric mucosa. The J-scoring method was used to assess severity of macroscopically visible changes in the mucous membrane. According to this method, the erosions were classified as follows: no erosions = 0; 0–1 mm in diameter = 1; 1–2 mm = 2; and greater than 2 mm in diameter = 3. The gastric index was the sum of these measured areas in each animal [24].

The histopathological examination of all stomach specimens was carried out in a blinded way by the experienced pathologist, who assessed independently the inflammation process and the damage of the gastric mucosa. The severity of the inflammation was assessed using a 0–3 scale (0—no inflammation, 1—mild inflammation, 2—moderate, and 3—severe inflammation). The severity of the damage of gastric mucosa was assessed using a 0–3 scale (0—no damage, 1—superficial erosion, 2—submucous ulceration, and 3—ulceration in *muscularis propria*). The sum of the inflammation and damage scores represented the cumulative microscopic gastric index.

### 2.9. Statistical Analysis

All experimental data are presented as mean values ± standard deviation (SD). Statistical differences between the studied parameters were analyzed using a one-way analysis of variance (ANOVA) and the NIR Fischer post hoc test. All statistical analyses were performed with Statistica v. 13.1 (Tibco Software, Palo Alto, CA, USA) with the statistical significance set at a *p*-value of <0.05.

## 3. Results

### 3.1. Tail Flick Test

**Figure 5:**
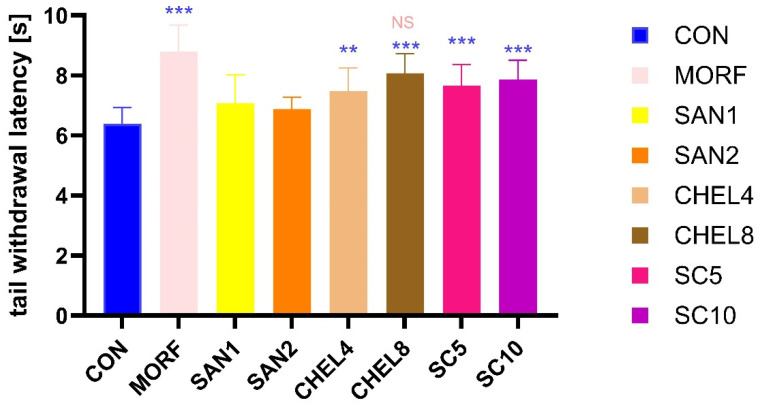
Effects of SC, SAN, and CHEL on the latency time during the tail flick test. Experimental groups: CON—the negative control group, MORF—animals receiving morphine 10 mg/kg, SAN1—animals receiving sanguinarine 1 mg/kg, SAN2—animals receiving sanguinarine 2 mg/kg, CHEL4—animals receiving chelerythrine 4 mg/kg, CHEL8—animals receiving chelerythrine 8 mg/kg, SC5—animals receiving sanguinarine–chelerythrine fraction of *C. chinensis* 5 mg/kg, SC10—animals receiving sanguinarine–chelerythrine fraction of *C. chinensis* 10 mg/kg. **—*p* < 0.01 vs. CON, ***—*p* < 0.001 vs. CON, NS—not significant vs. MORF.

There was a significant prolongation of the tail withdrawal latency in the groups receiving morphine, chelerythrine, and the sanguinarine–chelerythrinel fraction of *C. chinensis* compared to the negative control group (MORF: 8.79 ± 0.89 s, CHEL4: 7.48 ± 0.77 s, CHEL8: 8.08 ± 0.65 s, SC5: 7.66 ± 0.71 s, and SC10: 7.87 ± 0.65 s vs. CON: 6.39 ± 0.54 s, respectively) (CON—the negative control group, MORF—animals receiving morphine 10 mg/kg, SAN1—animals receiving sanguinarine 1 mg/kg, SAN2—animals receiving sanguinarine 2 mg/kg, CHEL4—animals receiving chelerythrine 4 mg/kg, CHEL8—animals receiving chelerythrine 8 mg/kg, SC5—animals receiving the sanguinarine–chelerythrine fraction of *C. chinensis* 5 mg/kg, and SC10—animals receiving the sanguinarine–chelerythrine fraction of *C. chinensis* 10 mg/kg), whereas there was no significant prolongation of tail flick latency in groups receiving sanguinarine (SAN1: 7.08 ± 0.04 s and SAN2: 6.88 ± 0.40 s). Additionally, no significant difference in the tail withdrawal latency between the MORF and CHEL8 groups was detected (Figure 5).

**Figure 6:**
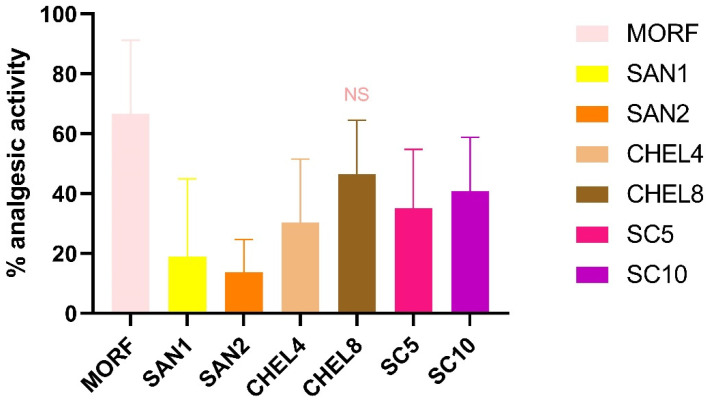
Percentage of analgesic activity of SC, SAN, and CHEL during the tail flick test. Experimental groups: MORF—animals receiving morphine 10 mg/kg, SAN1—animals receiving sanguinarine 1 mg/kg, SAN2—animals receiving sanguinarine 2 mg/kg, CHEL4—animals receiving chelerythrine 4 mg/kg, CHEL8—animals receiving chelerythrine 8 mg/kg, SC5—animals receiving sanguinarine–chelerythrine fraction of *C. chinensis* 5 mg/kg, SC10—animals receiving sanguinarine–chelerythrine fraction of *C. chinensis* 10 mg/kg. NS—not significant vs. MORF.

An analysis of the percentage of analgesic activity (Figure 6) revealed that there is no significant difference in analgesic activity between morphine 10 mg/kg and chelerythrine 8 mg/kg (66.6% ± 24.6% vs. 46.7% ± 18.1%). In all the other groups, significantly lower analgesic activity was detected. The percentage of the analgesic activity of sanguinarine (1 and 2 mg/kg), chelerythrine 4 mg/kg, and the sanguinarine–chelerythrine fraction of *C. chinensis* 5 and 10 mg/kg was significantly lower than in the animals receiving morphine 10 mg/kg (SAN1: 19.0% ± 26.0%, SAN2: 13.7% ± 11.1%, CHEL4: 30.3% ± 21.2%, SC5: 35.1% ± 19.7%, and SC10: 40.9% ± 17.9%, respectively).

### 3.2. Formalin Test

**Figure 7:**
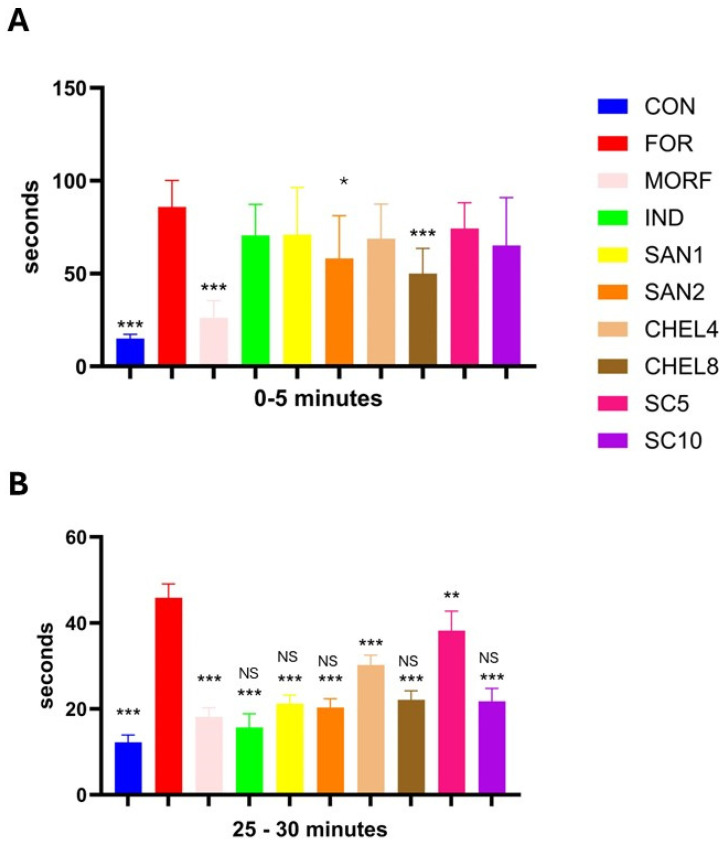
Effects of SC, SAN, and CHEL on the pain reaction (the duration of licking, flinching, shaking, and biting in seconds) during the neurogenic ((**A**)—early; 0–5 min) and inflammatory ((**B**)—late; 25–30 min) phase of the nociceptive reaction in the formalin test. Experimental groups: CON—the negative control group, FOR—the positive control group, MORF—animals receiving morphine 10 mg/kg, IND—animals receiving indomethacin 10 mg/kg, SAN1—animals receiving sanguinarine 1 mg/kg, SAN2—animals receiving sanguinarine 2 mg/kg, CHEL4—animals receiving chelerythrine 4 mg/kg, CHEL8—animals receiving chelerythrine 8 mg/kg, SC5—animals receiving the sanguinarine–chelerythrine fraction of *C. chinensis* 5 mg/kg, SC10—animals receiving the sanguinarine–chelerythrine fraction of *C. chinensis* 10 mg/kg. *—*p* < 0.05 vs. FOR, **—*p* < 0.01 vs. FOR, ***—*p* < 0.001 vs. FOR, NS—not significant vs. MORF.

Formalin significantly prolonged the pain reaction in both phases, the neurogenic and the inflammatory one, during the formalin test compared to the control group that received a saline injection (87 ± 13 s vs. 14 ± 3 s and 45 ± 3 s vs. 12 ± 2 s, respectively). Only morphine 10 mg/kg, chelerythrine 8 mg/kg, and sanguinarine 2 mg/kg ameliorated the pain reaction in the neurogenic phase of the formalin test (MORF: 26 ± 7 s, CHEL8: 50 ± 9 s, and SAN2 58 ± 14 s). However, the analgesic response to morphine was significantly more pronounced than the response to the investigated alkaloids. In the inflammatory phase of the formalin test, a significant reduction in pain response was observed in all groups receiving the investigated substances (CHEL, SAN, and SC), as well as in the groups receiving morphine and indomethacin as reference drugs (Figure 7).

### 3.3. MMP-9 and TNFα in Paw Homogenates

Formalin injection induced the inflammation of paw tissues, leading to an increased concentration of MMP-9 (Matrix Metalloproteinase-9) and TNFα (tumor necrosis factor α) in paw homogenates (Table 1). This was ameliorated by the administration of indomethacin, a reference anti-inflammatory drug. The reduction in inflammatory response was also observed in animals receiving tested alkaloids and the investigated fraction of *C. chinensis* extract. Additionally, there was no significant difference in the inhibitory effect on MMP-9 and TNFα secretion between the IND group and groups receiving higher CHEL, SAN, and SC doses.

### 3.4. Histopathological Assessment of Gastric Mucosa

**Figure 8:**
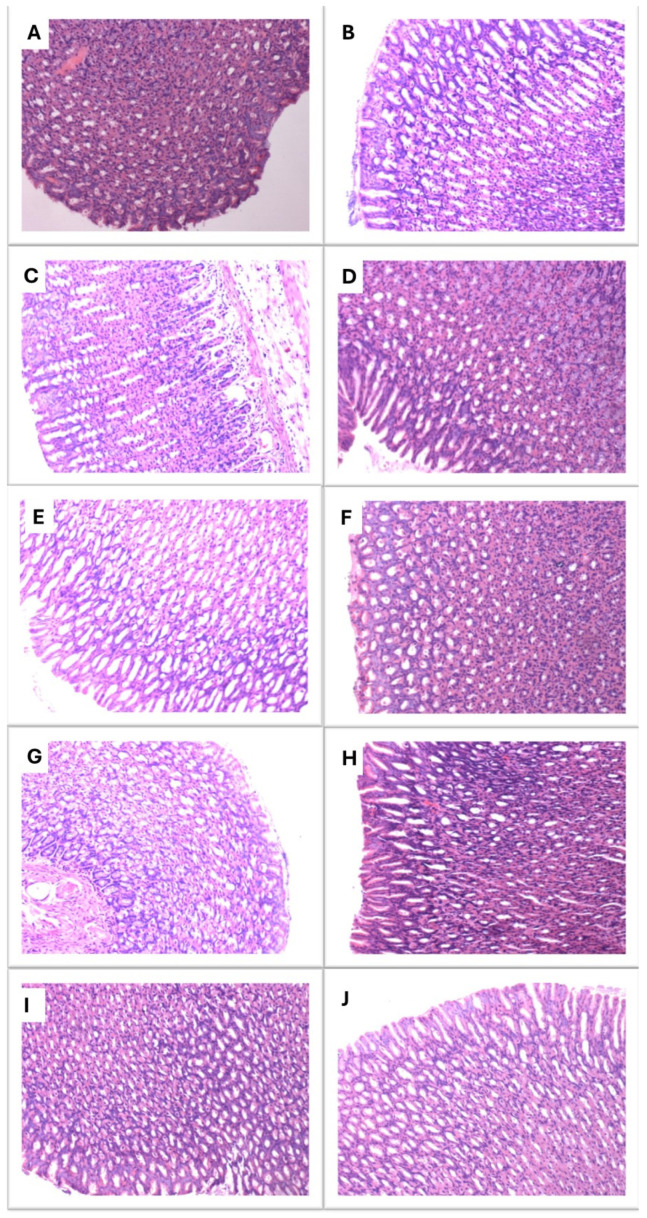
Microscopic examination of gastric mucosa after hematoxilin–eosin (H&E) staining (×100). Experimental groups (*n* = 10): (**A**) negative control group (CON); (**B**) positive control group (FOR); (**C**) group receiving indomethacin 10 mg/kg (IND); (**D**) group receiving morphine 10 mg/kg (MORF); (**E**) group receiving sanguinarine 1 mg/kg (SAN1); (**F**) group receiving sanguinarine 2 mg/kg (SAN2); (**G**) group receiving chelerythrine 4 mg/kg (CHEL4); (**H**) group receiving chelerythrine 8 mg/kg (CHEL8); (**I**) group receiving sanguinarine–chelerythrine fraction of *C. chinensis* 5 mg/kg (SC5); (**J**) group receiving sanguinarine–chelerythrine fraction of *C. chinensis* 10 mg/kg (SC10). In MORF group (**D**) and CHEL8 group (**H**), generally, normal mucosa showing mild inflammation is detected. In indomethacin-receiving animals (**C**), moderate inflammation in mucosa is present. In pictures (**A**,**B**,**E**–**G**,**I**,**J**), normal mucosa with no inflammation is observed.

The assessment of the gastric safety profile of the investigated compounds was based on the macroscopic and microscopic examinations of the stomachs. Macroscopic and microscopic examinations of the gastric mucosa did not reveal any significant gastric injuries in the experimental groups (Table 2 and Figure 8).

## 4. Discussion

In this study, we assess the antinociceptive effects of sanguinarine chloride (SAN), chelerythrine chloride (CHEL), and the sanguinarine–chelerythrine fraction of *Coptis chinensis* extract (SC) in a rat model. Our goal is not only to evaluate the effectiveness of the above compounds but also to compare their analgesic properties and safety of use. The most important findings are that, in the case of the groups receiving chelerythrine and the sanguinarine–chelerythrine fraction of *C. chinensis* extract, there was a significant prolongation of the tail withdrawal latency compared to the negative control group in the tail flick test, and also, there was no relevant difference in analgesic activity in the group receiving a higher dose of chelerythrine and morphine. Moreover, in the formalin test, we showed that in both of the mentioned groups—MORF and CHEL8—there was a significant reduction in the pain reaction cumulative time in the early, neurogenic phase, while in the late inflammatory phase, we observed such a decrease in several research groups, especially those receiving higher doses of the tested substances—sanguinarine, chelerythrine, and the sanguinarine–chelerythrine fraction of *C. chinensis* extract.

To our knowledge, this is the first experiment to compare the analgesic and anti-inflammatory activity of *C. chinensis* extract and the two alkaloid components administered separately. The current work is a continuation of our previous one, in which we proved, among others, that the sanguinarine–chelerythrine fraction of *C. chinensis* extract possesses anti-inflammatory activity comparable to indomethacin and does not cause damage to the gastric mucosa [21]. In the reported study, we try to answer the question of whether a natural mixture of alkaloids from the extract has a stronger or weaker effect than the corresponding doses of the substances administered separately and to assess the dose–effect dependency. In addition to assessment of anti-inflammatory activity in the current study, we assess the analgesic effect of the investigated agents, which has not been studied before.

In the tail flick test, we observed that the reaction time to pain stimuli in the form of a heat source was significantly longer in animals from the MORF, CHEL8, SC5, and SC10 groups compared to the CON control group. The substances administered to the animals, morphine 10 mg/kg, chelerythrine 8 mg/kg, and *C. chinensis* extract at both tested doses, i.e., 5 mg/kg and 10 mg/kg, respectively, showed a relevant analgesic effect, substantially extending the tail withdrawal latency. We also obtained a similar result, although to a slightly lesser degree, in the group receiving a lower dose of chelerythrine (4 mg/kg). Nevertheless, the above results can be summarized with a conclusion that in all research groups receiving chelerythrine or the extract, regardless of the dose, we obtained a statistically significant extension of the tail withdrawal latency, confirming the promising antinociceptive potential of the tested substances. The tail withdrawal latency time, especially in the CHEL8 and SC10 groups, was prolonged by about 1.5 s compared to the control group. With the average time for the CON group being 6.39 s, they see an increase in latency time of 26.4% and 23.2%, respectively. The most positive result in such a comparison was certainly obtained in the MORF group—an extension of almost 2.5 s, i.e., 37.6%.

At the same time, in both research groups receiving sanguinarine, SAN1 and SAN2 (1 mg/kg and 2 mg/kg), we did not note a significant result, which, at first glance, may indicate that of both components of the extract fraction, it is chelerythrine that is the more influential one in the context of the analgesic effect. However, it is worth remembering that the doses of both active substances administered separately differ four times (1 mg/kg and 2 mg/kg for sanguinarine and 4 mg/kg and 8 mg/kg for chelerythrine); therefore, a clear comparison of the activity of both compounds is impossible to make. The use of the above doses of mono-administered substances results from the composition of the extract, in which both compounds occur approximately in the proportion 1 + 4, and our attempt to precisely determine the properties of the extract, which is the basic research material in this study.

Due to the application of such proportions, we could observe further interesting results. Firstly, the latency time in the CHEL4 group (corresponding to a lower dose of the extract) was slightly shorter than in the SC5 group, but in the case of the CHEL8 group, it was longer than in the SC10 group. Additionally, in the SAN1 group, the latency time was longer than in the SAN2 group. This may suggest a decreasing role of sanguinarine in obtaining an analgesic effect with an enhancement in the dose of the extract. This is, of course, a far-reaching conclusion based on the available data, but it is certainly a hypothesis worth further exploring. Secondly, the antinociceptive effect of the extract was not the sum of the impacts of sanguinarine and chelerythrine, and simply adding the results obtained for both substances administered separately did not translate into the effect of the corresponding doses of the extract. This was an expected result, but we achieved its specific experimental validation.

Translating the above observations into an analysis of analgesic activity, three research groups, MORF, CHEL8, and SC10, were characterized by the highest average values of analgesic activity, 66.6%, 46.7%, and 40.9%, respectively. And even though the result of the SC10 group was relatively high, it was statistically different from MORF. There was no relevancy in the comparison of MORF vs. CHEL8. It is therefore justified to conclude that the analgesic activity of both groups is comparable, i.e., the use of chelerythrine at a dose of 8 mg/kg gives, to some extent, a similar antinociceptive effect to that of morphine at a dose of 10 mg/kg. The *C. chinensis* extract, although weaker in comparison, provides grounds for further analysis with the potential to obtain promising results. The findings presented above may suggest the extract’s dose dependency, so it can be assumed that increasing the dose of the extract may enhance its analgesic activity and obtain an effect at least comparable to morphine in 10 mg/kg. However, these are only the authors’ speculations that require further confirmation.

One of the probable mechanisms of the analgesic effect of the tested alkaloids is the impact on nociceptive signals by modulating glycine transporters. Jursky et al. reported that chelerythrine and sanguinarine selectively inhibit the glycine transporter GlyT1 with similar activity in the low micromolar range. GlyT1 inhibition by sanguinarine was irreversible, whereas the chelerythrine effect washed out over time [20]. Another mechanism—the inhibition of the microglia and p38 MAPK signaling pathway activation—was confirmed in neuropathic pain studies [18,19].

Outcomes corresponding to ours were obtained in several studies but were mainly concerned with chelerythrine. Wen et al. confirmed the attenuation of pertussis toxin (PTX)-induced thermal hyperalgesia by i.a. chelerythrine and associated this effect with protein kinase C inhibition. Additionally, they observed that chelerythrine could alleviate the PTX-induced reduction in morphine analgesia [25]. Similar results, including a complete reversion of morphine antinociceptive tolerance by a small dose of chelerythrine pretreatment, were described in other reports [26,27,28]. Sanguinarine, in turn, was frequently mentioned as an antimicrobial agent [29,30,31,32,33]. Our assumption about the potential analgesic activity of such a combination of alkaloids is, to a certain extent, reflected in research on another herb. Zanthoxyli Radix, a dried root of *Zanthoxylum nitidum*, is a raw material of plant origin, containing both sanguinarine and chelerythrine, used in traditional Chinese medicine as an analgetic [34,35].

In the formalin test, we divided the analysis into two phases: early neurogenic, during which we observed the reaction to pain in the form of licking, flinching, shaking, and biting during the first 5 min after formalin application, and late inflammatory, in which we made similar observations during the 25–30 min from application. In the early phase, we noted a relevant reduction in the pain reaction cumulative time compared to the group receiving only formalin in the MORF, CHEL8, and CON groups. We also observed relatively noticeable shorter reaction times in the SAN2 and SC10 groups, but this did not translate to statistically significant differences. We can, therefore, conclude that there is consistency in the results, where positive outcomes, more or less comparable to the usage of morphine, are obtained especially in the CHEL8 group, as well as SC10, where also, once again, the result is more favorable than in the case of SC5.

In the late inflammatory phase, the cumulative reaction time in each of the research groups was significantly shorter compared to that in the FOR group, and in the case of each of the analyzed substances—regardless of whether it was sanguinarine, chelerythrine or *C. chinensis* extract—the result was noticeably more favorable in groups receiving a higher dose of the tested compound. The largest positive difference (i.e., the greatest reduction in the pain reaction time) in the comparison between a higher and lower dose of the tested substance was noted in the case of the extract. These results are consistent with our hypothesis about the extract’s dose–effect dependency, where, once again, the higher dose turned out to be more effective, and also reflect the outcomes in the mono-alkaloid research groups. In this phase of pain reaction, we did not observe any significant differences between the MORF, IND, SAN1, SAN2, CHEL8, and SC10 groups. This may indicate that the analgesic effect of sanguinarine, chelerythrine, or an extract containing both of these alkaloids is directly related to the anti-inflammatory effect of these substances. This statement is reflected in various other reports [36,37,38,39] and is also consistent with our previous study [21].

In the TNFα assay, we observed a relevant increase in the positive control group (FOR) compared to the negative control (CON). Of the tested compounds, indomethacin showed the most effective reduction in the TNFα level. However, in all groups receiving the tested alkaloids, regardless of the dose and compound, we noted significantly lower levels of TNFα than in the FOR group to varying degrees. Once again, higher doses of the tested substances were characterized by greater efficacy with outcomes not statistically different from the IND group. The lowest level of TNFα in the groups receiving alkaloids was observed in the SC10 group, which may suggest, at least in part, the beneficiary effect resulting from the cumulative action of both sanguinarine and chelerythrine. There are several reports of the beneficial impact of *C. chinensis* derivatives on TNF-α levels, often involving traditional Asian medicine products that include *Coptis*. However, this positive effect in all studies is rarely directly linked to sanguinarine or chelerythrine action [40,41,42,43,44].

TNFα is a multifunctional cytokine with a strong pro-inflammatory effect involved in the pathogenesis of various diseases. It has an impact on diverse developmental and immunological processes, including inflammation, differentiation, cell communication, lipid metabolism, and apoptosis [45,46,47]. TNFα is considered a key factor in the initiation and maintenance of neuropathic pain [48]. It triggers a cytokine storm and boosts a cascade of other cytokines in pain-related pathways, thereby inducing and modulating neuropathic pain by facilitating peripheral (i.e., by sensitizing nociceptors) and central sensitization [49,50]. It also plays an important role in regulating pain communication between the immune system and the brain [51].

In the assessment of matrix metalloproteinase-9, we observed a relevant increase in the positive control group compared to the negative control. In all groups receiving the tested alkaloids or the extract, the level of MMP-9 was significantly lower compared to the FOR group. Interestingly, enzyme concentrations were lower in groups with higher doses of sanguinarine and chelerythrine administered separately (which seems to be a logical consequence), but in the SC10 group, the effect of reducing the MMP-9 level was less than in the SC5 group. In our previous report on the extract, we also noted a decrease in the MMP-9 level compared to the group treated with a pro-inflammatory factor, in that case, carrageenan. One of the assumptions of the current study was to expand the research by including groups receiving the tested alkaloids separately to perform a comparative analysis. The results obtained in this assay foreclose to draw a clear conclusion and also disturb the previously stated hypothesis of the dose dependency of the extract. Further research is needed to thoroughly verify and obtain repeatable results. However, it is worth noting that in all research groups receiving alkaloids, regardless of whether the substances were administered separately or in the form of an extract containing both tested substances and regardless of the dose used, the level of MMP-9 did not differ statistically from the level in the IND group. This means that the potential of these substances in the late inflammatory phase of the pain reaction is comparable to the effect of indomethacin, one of the strongest anti-inflammatory drugs from the NSAID group.

Matrix metalloproteinase-9 is an endopeptidase, also known as gelatinase B or type IV collagenase, that plays a substantial role in trauma reaction after tissue injury, among others, through interaction with various cytokines and growth factors, including TNFα. It is particularly important in the case of neuropathic pain, mechanical allodynia, thermal hyperalgesia, inflammation, and oedema in many different tissues [52,53]. Increased MMP-9 may contribute to the degradation of various protein components of the extracellular matrix, including type IV collagen and gelatin, through a wide range of physiological and pathophysiological processes involving tissue remodeling [54,55,56]. The evaluation of MMP-9 levels, one of the important pro-inflammatory factors, harmonizes with the outcomes of pain reaction analysis in the late inflammatory phase, suggesting that the analgesic activity of the *C. chinensis* extract containing the sanguinarine–chelerythrine fraction results mainly from the anti-inflammatory properties of the assessed alkaloids. Interestingly, the anti-inflammatory or analgesic effects of the derivatives acquired from *C. chinensis*, e.g., dried rhizome or extract, were often associated primarily with the activity of another alkaloid present in this herb, berberine [13,57,58,59,60,61]. Our promising results shed new light on this issue, proving that sanguinarine and chelerythrine may be important constituents of these effects.

During our experiment, we did not observe any significant damage to the gastric mucosa, both macroscopically and microscopically. We also obtained similar results in our previous study on the extract’s properties [21]. Many commonly used analgesic drugs belong to the group of NSAIDs, which, especially in the form of COX-1 inhibitors, may harm the gastric mucosa. Therefore, searching for agents comparable in efficacy but safer to use is more than necessary. The sanguinarine–chelerythrine fraction of *C. chinensis* extract may be a promising alternative to this approach. Moreover, Lin et al. reported that sanguinarine is not only harmless for the gastrointestinal mucosa but may even effectively reverse the inflammatory lesions induced by indomethacin in rats’ small intestines [54]. In turn, Shi et al. stated that sanguinarine may improve intestinal health by enhancing intestinal antioxidant ability, alleviating intestinal barrier damage, and ameliorating intestinal microbiota homeostasis, which was confirmed in a grass carp model [22].

Regarding the greatest limitation of our study, we consider it to be the lack of the application of a third, larger dose of the extract. However, this is a conclusion made a posteriori based on the results obtained, which showed that a larger dose of the extract would possibly allow us to achieve outcomes fully comparable to the effects of two positive control compounds, indomethacin and morphine, while maintaining safety for the gastric mucosa.

## 5. Conclusions

Pain is an important therapeutic problem in modern medicine and the application of nonsteroidal anti-inflammatory drugs and opioid analgesics is often associated with significant side effects. In our study, we found that the sanguinarine and chelerythrine fractions of *C. chinensis* possess significant analgesic activity. To the best of our knowledge, this is the first study comparing the analgesic and anti-inflammatory activity of the sanguinarine–chelerythrine fraction from *C. chinensis* extract and two alkaloid components administered separately. Our results confirmed the analgesic effects of chelerythrine and the sanguinarine–chelerythrine fraction, with chelerythrine analgesic activity comparable to morphine. Additionallym all investigated substances exerted significant anti-inflammatory activity without concomitant gastrotoxicity. Summing up, sanguinarine–chelerythrine from *C. chinensis* is a valuable candidate for further research on new analgesic therapeutic options. Regarding the greatest limitation of our study, we consider it to be the lack of the application of a third, larger dose of the extract. However, this stems from the fact that the reported study was a continuation of our earlier research.

## Figures and Tables

**Table 1:** Effects of SC, SAN, and CHEL on MMP-9 and TNFα concentration in paw homogenates.

	CON	FOR	MORF	IND	SAN1	SAN2	CHEL4	CHEL8	SC5	SC10
MMP-9 [pg/mL]	536.3 ± 84.8 **	805.9 ± 160.3 ^^	676.0 ± 131.2	551.9 ± 117.7 **	552.2 ± 136.4 **^, NS^	528.6 ± 83.7 **^, NS^	572.8 ± 125.2 *^, NS^	526.4 ± 74.3 **^, NS^	537.9 ± 172.6 **^, NS^	592.3 ± 58.6 *^, NS^
TNFα [pg/mL]	29.72 ± 12.39 *	43.09 ± 9.91 ^	34.11 ± 12.86	11.75 ± 2.81 ***	24.07 ± 7.04 **	19.93 ± 3.42 ***^, NS^	30.44 ± 8.58 *	24.37 ± 4.91 **^, NS^	23.30 ± 3.89 **	19.04 ± 2.70 ***^, NS^

Experimental groups: CON—the negative control group, FOR—the positive control group, MORF—animals receiving morphine 10 mg/kg, IND—animals receiving indomethacin 10 mg/kg, SAN1—animals receiving sanguinarine 1 mg/kg, SAN2—animals receiving sanguinarine 2 mg/kg, CHEL4—animals receiving chelerythrine 4 mg/kg, CHEL8—animals receiving chelerythrine 8 mg/kg, SC5—animals receiving sanguinarine–chelerythrine fraction of *C. chinensis* 5 mg/kg, SC10—animals receiving sanguinarine–chelerythrine fraction of *C. chinensis* 10 mg/kg, MMP-9—Matrix Metalloproteinase-9, and TNFα—tumor necrosis factor α. Data are presented as mean ± SD. ^—*p* < 0.05 vs. CON, ^^—*p* < 0.01 vs. CON, *—*p* < 0.05 vs. FOR, **—*p* < 0.01 vs. FOR, ***—*p* < 0.001 vs. FOR, ^NS^—*p* > 0.05 vs. IND.

**Table 2:** Effects of sanguinarine, chelerythrine and sanguinarine–chelerythrine fraction of *C. chinensis* extract on gastric mucosa. Indomethacin and morphine are used as reference drugs.

Group	Macroscopic Evaluation	Microscopic Evaluation (H&E Staining)		
	Gastric Index	Inflammation Score (0–3)	Gastric Mucosa Damage Score (0–3)	Cumulative Microscopic Gastric Index (0–6)
CON	0.0 ± 0.0	0.500 ± 0.548 **	0.0 ± 0.0	0.500 ± 0.548 **
FOR	0.0 ± 0.0	0.500 ± 0.535 **	0.0 ± 0.0	0.500 ± 0.535 **
IND	0.0 ± 0.0	1.500 ± 0.577 ^^	0.0 ± 0.0	1.500 ± 0.577 ^^
MORF	0.0 ± 0.0	1.000 ± 0.000	0.0 ± 0.0	1.000 ± 0.000
SAN1	0.0 ± 0.0	0.833 ± 0.408 *	0.0 ± 0.0	0.833 ± 0.408 *
SAN2	0.0 ± 0.0	0.833 ± 0.408 *	0.0 ± 0.0	0.833 ± 0.408 *
CHEL4	0.0 ± 0.0	0.333 ± 0.516 ***	0.0 ± 0.0	0.333 ± 0.516 ***
CHEL8	0.0 ± 0.0	0.833 ± 0.408 *	0.0 ± 0.0	0.833 ± 0.408 *
SC5	0.0 ± 0.0	0.500 ± 0.548 **	0.0 ± 0.0	0.500 ± 0.548 **
SC10	0.0 ± 0.0	1.000 ± 0.632	0.0 ± 0.0	1.000 ± 0.632

Effects of SC, SAN, and CHEL on the latency time during the tail flick test. Experimental groups: CON—the negative control group, MORF—animals receiving morphine 10 mg/kg, SAN1—animals receiving sanguinarine 1 mg/kg, SAN2—animals receiving sanguinarine 2 mg/kg, CHEL4—animals receiving chelerythrine 4 mg/kg, CHEL8—animals receiving chelerythrine 8 mg/kg, SC5—animals receiving the sanguinarine–chelerythrine fraction of *C. chinensis* 5 mg/kg, SC10—animals receiving the sanguinarine–chelerythrine fraction of *C. chinensis* 10 mg/kg. H&E—hematoxylin–eosin. The J-scoring method is used to assess the severity of macroscopic gastric damage. It classified the erosions as follows: no erosions = 0; 0–1 mm in diameter = 1; 1–2 mm = 2; greater than 2 mm in diameter = 3. The cumulative macroscopic gastric index is defined as the sum of these measured areas in each animal [24]. The microscopic evaluation assesses independently the inflammation process and the damage of gastric mucosa. The severity of the inflammation is assessed using a 0–3 scale (0—no inflammation, 1—mild inflammation, 2—moderate, and 3—severe inflammation). The severity of the damage of gastric mucosa is assessed using a 0–3 scale (0—no damage, 1—superficial erosion, 2—submucous ulceration, 3—ulceration in *muscularis propria*). The cumulative microscopic gastric index is defined as the sum of the inflammation and damage score. Data are presented as mean ± SD. *^^—p* < 0.01 vs. CON; *—*p* < 0.01 vs. IND, **—*p* < 0.01 vs. IND, and ***—*p* <0.001 vs. IND.

## Data Availability

Data are contained within the article.
